# Talar body fracture combined with medial malleolar fracture: a case report and literature review

**DOI:** 10.11604/pamj.2019.32.57.6166

**Published:** 2019-01-31

**Authors:** Kamal Lahrach, Khalid Ibn El Kadi, Amine Marzouki, Fawzi Boutayeb

**Affiliations:** 1 Department of Orthopedic Surgery A, University Hospital Hassan II, Faculty of Medicine and Pharmacy, Fès, Morocco

**Keywords:** Talar body, medial malleolus, sagittal fracture, osteosynthesis

## Abstract

Fracture of the talar body is rare, it reaches articular congruence and determines the ankle's functional prognosis. The authors report a case of a combination of a sagittal talar body fracture with fracture of the medial malleolus in a 52-year-old following a road traffic accident. The association talus body fracture with a medial malleolus fracture is exceptional. Stable internal fixation can yield good functional results.

## Introduction

The incidence of talar fractures is lower (0.3%) and talar body fractures are the most frequent (60% of all talar fractures) [1]. A combination of a sagittal talar fracture and medial malleolar fracture seems to be very rare. Sporadic cases have been reported with unpredictable outcome, severe complications dominated by aseptic necrosis and osteoarthritis. However, association of medial malleolus fracture would preserve vascular branches from the deltoid ligament and irrigating the slope and therefore, limit the risk of necrosis. We discuss a case of this association in the literature review.

## Patient and observation

**Figure 1:**
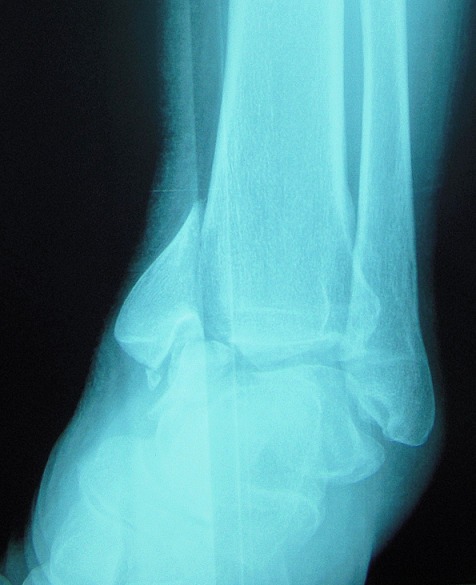
Radiography on admission

**Figure 2:**
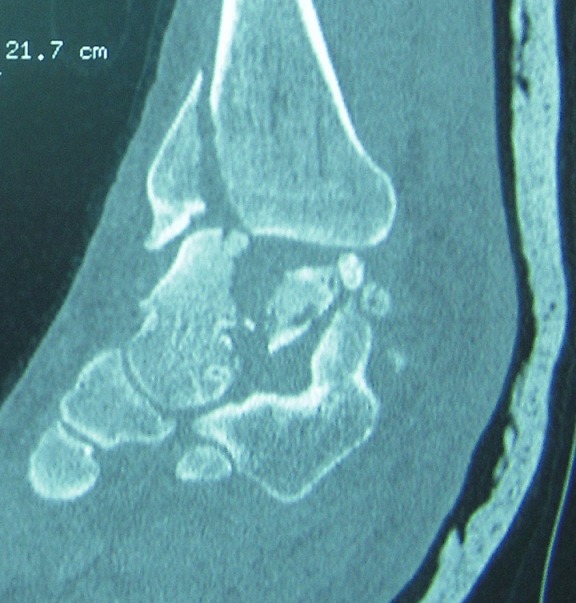
Computed tomogram on admission

**Figure 3:**
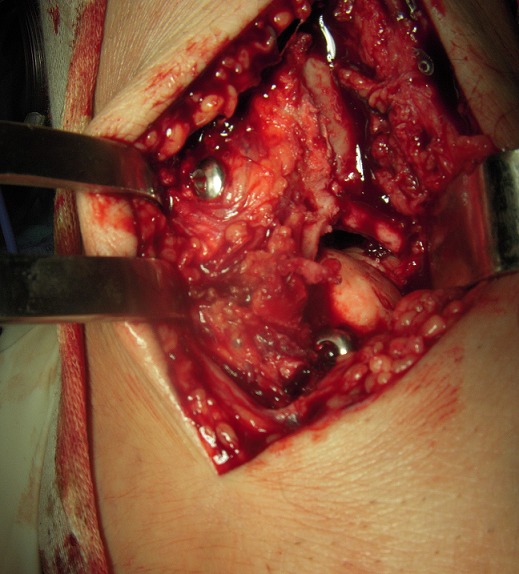
Intraoperative photography shows before and after fixation screw

**Figure 4:**
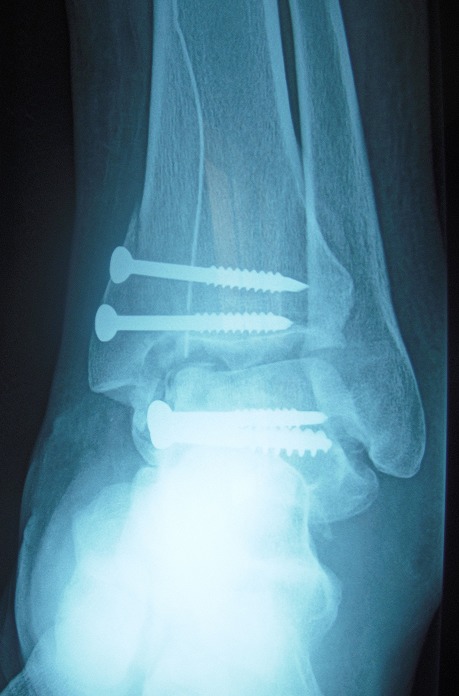
Postoperative radiological control with a little fragment of fracture anterior to the body

MKL, 52 year-old man, official, without medical history, was the victim of a traffic accident (motor vehicle accident) driving home blunt trauma of the right ankle with pain and overall functional impairment. The mechanism associated dorsiflexion, inversion and axial compression. Clinical examination revealed a large inflammatory ankle. The medial malleolus was very painful on palpation. There was no open wound, or neurovascular disorders. Radiographic and CT scan showed an ankle fracture oblique to the medial malleolus and a sagittal talar body fracture (Figure 1, Figure 2). A first reduction was performed by emergency maneuver the hard boot and abduction. The treatment consisted on surgery the next day under spinal anesthesia. Through the fracture of the medial malleolus, talus fracture was reduced and stabilized by two cancellous screws. Then the medial malleolus was reduced and fixed by two screws (two malleolar screws) (Figure 3). Contention by plaster boot was made for a period of six weeks. Postoperative radiological control was satisfactory (Figure 4). After removal of the boot, functional rehabilitation was established, and the vertical integration with full support was authorized for three months postoperatively. Six months after surgery, the patient shows no discomfort or pain, he gets back his work without any complaint (but 6 months is too short to eliminate osteonecrosis and osteoarthritis).

## Discussion

Fractures of the talus are rare (0.3% of all fractures), they are often associated with fractures of the neck of the foot, including a broken ankle in 26 % of cases [1-5]. The combination of a body sagittal fracture of the talus and the medial malleolus is exceptional. Few cases have been reported [4, 6-8]. Fractures of the talus follow an axial compression between the talus and the calcaneus tibiofibular mortise associated with forced dorsiflexion of the foot (5). This mechanism occurs when high-energy accidents: airplane crashes (rudder), car, motorcycle, fall from high altitude [3, 5, 9]. The existence of a medial malleolar fracture requires, in addition to the axial compression of the dorsiflexion a forced supply of the foot [5]. The bank then comes up against the mortise ceiling to superomédial angle and it thus occurs concomitantly a vertical fracture of the talus and the medial malleolus [5, 6]. Thus lesions located on the medial side of the body of the talus require supination, so the lateral side of the lesions occurs in fractures with pronation and external rotation [10]. Each fracture of the talus described in our patient is classified C according to the classification of Sneppen *et al.* [10] and B according to the AO classification. But these classifications are incomplete, because they don't consider the associated malleolar fractures [6, 11]. Vascularization of the slope is precarious; it is influenced by three arteries: posterior tibial, anterior tibial and perforating branch of the peroneal artery. The medial half of the body of the talus is vascularized by the collateral of the posterior tibial artery from hich deltoid artery travels in the deltoid ligament [12]. The presence of an ankle fracture reduces the risk of developing post- traumatic necrosis of the talus preserving capsuloligamentary between the fragments of the talus and fractured malleolus [3, 4, 6, 13]. But the risk of post- traumatic necrosis depends also of soft tissue lesions, vascular damage, the force of trauma, and therapeutic quality [14, 15]. It occurs in 40 % of cases [10, 16]. Post-traumatic osteoarthritis is unpredictable; it is directly influenced by the importance of trauma and progressive complications [17]. Surgical treatment is required a. The reduction in open hearth articular surfaces must be complete and anatomical followed by internal fixation, is stable [14, 15, 18] by screwing or stay for the medial malleolus. The materials used are small screws or pins for slopes and cancellous screws or plug bracing for medial malleolus [18, 19]. The choice of incision is anteromedial, remote vascular pedicles [17]. The osteotomy of the medial malleolus, which is recommended to better expose the talus [20], is replaced by the fracture.

## Conclusion

Fracture of the talus body is rare. The association with a medial malleolus fracture is exceptional, but providential infact it protects vasculature fragments detached and offers for one day an anatomic reconstruction of the talus. A support with rapid restoration of articular congruence and stable internal fixation can yield a good functional outcome.

## Competing interests

The authors declare no competing interests.
